# Goal-Directed Health Care: Redefining Health and Health Care in the Era of Value-Based Care

**DOI:** 10.7759/cureus.1043

**Published:** 2017-02-21

**Authors:** James Mold

**Affiliations:** 1 Family and Preventive Medicine, University of Oklahoma HSC

**Keywords:** primary care, health, goal-directed health care, goals, prevention, quality of life, advance directives, growth and development, health definition, definition of health

## Abstract

Health care reform efforts have increasingly emphasized payment models that reward value (quality/cost). It seems appropriate, therefore, to examine what we value in health care, and that will require that we examine our definition of health. In spite of admonitions from the World Health Organization and others, our current health care system operates under the assumption that health represents the absence of health problems. While that perspective has led to incredible advances in medical science, it now may be adversely affecting value. Problem-oriented care is clearly one of the drivers of rising costs and it could be adversely affecting the quality of care, depending upon how quality is defined.

If we redefined health in terms of patient-centered goals, health care could be focused more directly on meaningful outcomes, reducing the number of irrelevant tests and treatments. Greater emphasis would be placed on prevention, meaningful activities, advance directives and personal growth and development. The role of patients within clinician-patient relationships would be elevated, strengthening therapeutic relationships. Reframing health in terms of health-related goals and directing the health care system to help people achieve them, could both improve quality and reduce costs. In the process, it could also make health care less mechanical and more humane.

## Introduction and background

We are about three decades into a concerted effort on the part of government and industry to reform our health care system, primarily because it is so expensive, but also because we have fallen behind other developed countries on most measures of population health. Recent improvement strategies have recognized the importance of primary care and have embraced the “Care Model,” developed by Ed Wagner and colleagues [1], and “Patient-Centered Medical Home" principles developed and adopted by the three major primary care professional associations [2]. These strategies require tighter adherence to evidence-based clinical practice guidelines, implementation of information technologies like electronic records, registries, and information exchange systems, and more effective teamwork.

Reformers are measuring the success of their efforts using the “Triple Aims,” a framework proposed by Donald Berwick, President Emeritus and Senior Fellow at the Institute for Healthcare Improvement and former Director of the Centers for Medicare and Medicaid Services. The Triple Aims are: (1) improving the quality of the care provided, (2) improving patient outcomes, and (3) reducing the cost of care [3]. Some have added a fourth Aim, improving the work life of health care providers.

Since the establishment of the Centers for Medicare and Medicaid Innovations center, a number of local and regional experiments are underway. Most include payment reforms designed to enhance primary care (e.g.: paying for care coordination and registries) and incentives to encourage clinicians to deliver evidence-based care. Many private experiments are also being conducted by health systems, insurance companies, and new health care businesses, including “Value-Based Purchasing” and “Direct Primary Care.” 

Nearly all of these experiments emphasize improving care processes and reducing costs. The outcomes being measured are typically intermediate outcomes like hemoglobin A1c and blood pressure, rates of utilization of emergency and hospital services, and patient satisfaction, outcomes that can be obtained over short periods of time from readily available data. Clinicians have raised concerns about these metrics, but the financial concerns are so prescient that there appears to be little appetite for debate or for truly disruptive ideas.

Nevertheless, before we go too much further down the path we are on, it could be important to examine our fundamental beliefs about health and the purpose of health care, particularly since our disease-oriented approach to health care may be one of the drivers of increasing costs [4]. In other words, before we begin incentivizing value-based care, we should make sure that we all patients, clinicians, payers, and policymakers agree on our definition of health and its implications for determining the value of health care. For purposes of this discussion, I will define value as it is typically defined in business as quality divided by cost (quality/cost).

## Review

### Ten ways problem-oriented care could be contributing to poor quality and rising cost

The admonition of the World Health Organization notwithstanding health care in the United States is clearly predicated on the notion that “health” represents the absence of disease. The assumption underlying the majority of health care interventions is that correcting abnormalities will restore health and thereby improve both length and quality of life. The problem-oriented approach has, of course, been extremely successful in many respects. However, now that we are really good at it, it may be contributing to both rising costs and undesirable outcomes.

Problem-oriented care could be reducing quality and increasing costs in at least ten different ways. First, because health is defined as the absence of problems, or a state of “normalcy,” costs would be expected to increase as the health care system gets better at detecting and correcting abnormalities. In fact, that appears to be what is happening. Nearly two-thirds of recent increases in health care costs can be attributed to the increasing prevalence and detection of diseases and to new tests and treatments [5].

It is also predictable that, with better tests and treatments, the parameters of normal will tighten over time. Based upon the law of diminishing returns, the cost required to bring people into alignment with those narrowing parameters will increase exponentially [6]. For example, there is no evidence that reducing the diagnostic threshold for diabetes from a fasting blood sugar of 140 mg/dl to 126 mg/dl has increased length or quality of life, but it has almost certainly increased health care costs [5]. Advances in genomics and genetic engineering threaten to bring this challenge to a head, raising more profound questions about whether seeking normalcy is even advisable [7]. Are we searching for the perfect (i.e., normal) genome?. If we were ever able to achieve that, would our species be able to survive? Doesn’t our survival depend upon diversity? [8].

Second, our population is aging. That has prompted an intense effort to define “normal aging,” for without adjusting normal parameters of health on the basis of age, problem lists quickly fill up with uncorrectable abnormalities. Even allowing for such adjustments, many physicians resist caring for older people because of the number, chronicity, and complexity of their health problems. Organizing care around a lengthy problem list often obscures the issues of greatest importance to an older person [9]. It is also depressing for both clinicians and patients to view aging as a downhill path characterized by poorer and poorer health.

Third, the interventions most often employed in problem-oriented care are naturally those directly related to the identified problems. Strategies that are not problem-specific, such as mindfulness, physical activity, and many alternative and integrative therapies, and primary preventive measures, such as healthy eating, hand-washing, and dental hygiene, are often neglected even when those strategies are potentially more impactful and less expensive than disease-specific strategies.

Furthermore, a problem-oriented approach encourages clinicians to identify the single underlying cause of each problem instead of viewing health challenges in context. For example, the cause of pneumococcal pneumonia is believed to be the bacterium, Streptococcus pneumoniae. That perception diminishes the importance of the factors that allowed sufficient numbers of bacteria to enter the lungs in the first place, prevented their clearance once they were there and permitted them to multiply and invade lung tissue. The failure to recognize contributing factors can lead to an overreliance on disease-specific treatments like antibiotics and an under-recognition of the importance of supportive and rehabilitative measures.

Fourth, the problem-oriented model lacks an effective way for clinicians and patients to decide whether and when intervention is required. For example, preventive services are currently recommended based on age, gender, and only a few other risk factors. There is no generally accepted method for prioritization based on each person’s overall risk profile or their outcome preferences. Calculations of impact are population based and hard to apply clinically. As a result, a significant number of people receive screening tests from which they are unlikely to benefit very much. 

When an individual chooses to bring her symptoms to the attention of a health care professional, the clinician will often try to identify their cause and prescribe treatment regardless of whether that will be best for the patient in the long-term. How many childhood ear infections have been treated too early with antibiotics, preventing the opportunity for an immune response, altering the upper respiratory biome, and encouraging even earlier treatment for the next episode? How many people with self-limited episodes of low back pain are now addicted to narcotics? The essential clinical question is, "Can this be treated?" rater than, "Should this be treated at this time?".

 Such directionless efforts to correct abnormalities can sometimes result in expensive and hazardous clinical cascades [10], a problem that has increased in frequency because of advanced imaging techniques and will increase further as DNA testing becomes, even more, routine [11]. The factors that fuel cascades, patient and clinician anxiety and the desire to gain control [12], also contribute to medical errors, now the third leading cause of death in the United States [13].

Fifth, from a problem-oriented perspective, death represents the failure of medical interventions. Large amounts of money are spent in the last weeks or months of life trying to keep people alive. Discussion of advance directives and end of life planning rarely occur until a terminal illness has been identified and referrals for hospice care are often delayed unnecessarily [14-15]. As a result, many people receive more aggressive and expensive medical care than necessary or desired [16].

Sixth, problem-oriented care is best suited to the care of people with acute solvable problems (e.g., infections, injuries, and surgical emergencies), situations in which context is not critical. However, an increasing number of people suffer from health problems that are multiple, chronic, incurable and linked to health behaviors and environmental factors. Forcing a round peg into a square hole inevitably causes damage. In this case, our approach has been to dichotomize risk factors (e.g., elevated weight, blood pressure, mood, alcohol consumption, etc.) and other variations between people, which fit most comfortably on bell-shaped curves, reconceptualizing them as diseases (e.g. obesity, hypertension, depression, alcoholism, etc.) to the tremendous benefit of clinical laboratories and pharmaceutical companies [17-18].

The Care Model, originally the Chronic Care Model, was developed to help primary care clinicians do a better job of managing patients with chronic illnesses. However, because it is being implemented within a problem-oriented framework, it has contributed to over-testing and over-treatment by encouraging the use of registries which generate disease-based, context-free task lists (e.g., lists of all patients due for an A1c test) managed by non-clinicians. This unintended effect of the model could potentially be addressed by fleshing out the meaning of “productive interactions.”

Seventh, the problem-oriented view of health and health care reinforces the tendencies of people to want to be “normal” and to compare themselves to others. That tends to increase anxiety and lead to unrealistic expectations. Perhaps the best published example of this phenomenon is the randomized trial of hypertension screening conducted in a workplace in Canada. Compared to those in “the screened and not told” group, those assigned to the “screened and advised” group subsequently exhibited increased absenteeism from work with no significant improvement in their blood pressures [19]. A different subgroup of people become overly dependent upon and sometimes even addicted to health care. Ivan llich called this “medicalization” [20].

Eighth, problem-oriented care can impede teamwork. Health care teams function best when their members agree upon and work towards the common goal, ideally goals endorsed by the patient. However, when the “goal” is actually a strategy, problem-solving, several undesirable things tend to happen. Physicians, because of their extensive training in medical problem-solving, exert undue influence [21]. In response, each discipline, in an attempt to gain stature, defines its own territory over which it claims expertise and control and the patient’s goals are often lost in the process. There is no real need for the various health professionals to communicate. Team dysfunction can result in reduced therapeutic creativity, duplication of effort, and fragmentation of care.

Ninth, when health care is problem-oriented adherence to recommendations suffers because problem resolution is at least one step removed from the goals that matter most to patients. I often wonder, for example, why anyone would be willing to take blood pressure medications every day for the rest of their life to manage their hypertension without knowing how much additional months of life they might gain by doing so?. Perhaps that is why adherence to some long-term medications is only about 50%.

Finally, the problem-oriented medical model weakens the clinician-patient relationship by creating an unequal relationship between clinicians and patients in which the clinician is clearly the expert. As diagnostic tests improve, clinicians rely less and less on the information provided by patients and standardization of treatment recommendations has reduced the perceived need to understand context. Because problems can be intellectually separated from the people who have them, a problem-oriented approach tends to dehumanize care. 

More insidiously, the problem-oriented approach has caused many of those involved in reforming the health care system to underestimate the importance of therapeutic relationships, when, in fact, therapeutic relationships and relationship-based strategies like reassurance, encouragement, supportive listening, reframing and therapeutic touch, are as important to health and healing as problem-solving [22].

### An alternative conceptualization of health and healthcare

The time has come to consider an alternative conceptualization of health and health care that better fits the way people actually think and behave. Most people view life as a journey characterized by amazing opportunities and tremendous challenges. They would likely define health as being able to get the greatest possible value from the trip. The things they value most are being able to do the things they enjoy and find meaningful for as long as possible meeting, overcoming and learning from the inevitable challenges along the way and then dying peacefully having had the opportunity to reach their full potential as human beings [23].

Those definitions of health and value suggest that health care should focus on four major health-related goals: 1) prevention of premature death and disability, 2) maintenance or improvement of quality of life, 3) maximization of personal growth and development and 4) preparation for a good death [24-26]. How might a goal-directed approach to health care improve care processes and outcomes while also reducing cost?.

Focusing directly on prevention of premature death and disability would clearly put greater emphasis on preventive strategies. Prevention has never fit comfortably into the problem-oriented model, and as a result, delivery of primary and secondary preventive services has always been less than optimal. Since each person has a unique set of risk factors, both individualization and prioritization of preventive strategies would be essential. With prevention as a goal rather than a strategy, primary, secondary, and tertiary strategies would be considered together. Because primary preventive strategies generally have a bigger impact on survival, they would be emphasized. That would necessitate closer collaboration with public and mental health professionals and community-based organizations.

Clinical discussions would focus on whether life prolongation was still a goal, circumstances under which it would no longer be a goal and the trade-offs each person would be willing to make between length and quality of life. Health risk assessment would assume greater clinical importance and techniques and accuracy would be improved. Longitudinal health planning would become a central feature of care.

A goal-directed approach to maintaining and enhancing quality of life (QOL) would require much greater individualization of care since QOL means something different to each person. That would create more equality in relationships between clinicians and patients since both parties would bring essential information to the relationship. Perhaps the best example of goal-directed care within the current healthcare system is occupational therapy. In fact, the optimal goal-directed primary health care team might include an occupational therapist.

Discussions would address typical, essential and desired activities and functional limitations and requirements related to them. Potential strategies would include maintenance, rehabilitative and preventive measures as well as environmental modifications and strengthening of social supports. 

The challenges that life poses create opportunities for physical, psychological and spiritual growth. And while growth can be difficult and even painful, most people value it more highly than almost anything else [27]. Deci and Ryan argue that human beings have three basic psychological needs, the need for connectedness, the need for autonomy and the need to feel competent [28]. Health care professionals have many opportunities to help people to meet these needs as they face many challenges that life presents to them. Viewing growth and development as a goal could direct more attention to this important component of health.

A goal-directed approach would encourage clinicians and patients to embrace death as an essential part of life. That would make it easier to broach the topics of advance directives and end of life planning well before the onset of a terminal illness, preventing at least some unwanted interventions during the final weeks of life [16]. Clinical discussions would involve both formal and informal advance directives and elucidation of values and preferences regarding end-of-life care.

Goals are qualitatively different from problems in at least three important ways. First, whereas problems are relatively value-neutral, goals are value-laden. It is much more difficult then to separate goals from the person who owns them. Second, goal-setting requires patient input, since the information required extends beyond scientific knowledge. That tends to equalize the relationship between medical expert and the person seeking assistance. Finally, goal-setting and goal achievement demand a greater level of shared understanding and mutual commitment within clinician-patient relationships simply because of the more personal nature of these activities. 

In contrast to problem-solving, goal-directed health care would be positive (optimistic), unlimited (no upper limit on health) and ageless (better health at any age). It would neither constrain nor inhibit scientific exploration and discovery, though research methods would need to evolve to account for individualization. Problem-solving would continue to be an important strategy, but there would be a logical and humane framework within which to apply it when indicated.

### Goal-directed care, quality, and cost

Whether goal-directed care would improve health care quality would depend upon the definition of quality. Currently, quality is defined by clinician adherence to disease-oriented guidelines and the degree to which patients achieve recommended levels of disease control. This conceptualization is similar to the definition of quality in manufacturing, in which the goal is product uniformity.

In 1988, G. E. Steffen, frustrated by years of quality assessment work, wrote in an article published in the Journal of the American Medical Association: “For the physician and patient, quality of medical care can be defined as that care that has the capacity to achieve the goals of the physician and the patient.” He also pointed out that, in most cases, the goals of health care actions are not documented in the medical record, making quality assessment impossible [29].

A fundamental assumption of goal-directed health care is that every person is unique. From that premise, it follows that high-quality health care should assure that each person is treated differently based upon their particular goals, challenges, resources, and preferences. For example, a substantial number of people with “hypertension” will not benefit from blood pressure reduction: those for whom life prolongation is no longer a goal, those with a limited life expectancy, those who will almost certainly die from something other than cardiovascular disease and those who are unable or unwilling to do what it would take to lower their blood pressure for the extended period of time required for them to benefit.

If we were to define quality of care as “care likely help patients achieve their health goals,” then goal-directed care would almost certainly be of higher quality than problem-oriented care for several reasons. Interventions would relate directly to meaningful outcomes and they would be prioritized based upon impact. By encouraging primary prevention, reducing irrelevant interventions and preventing clinical cascades, goal-directed care should also be safer. Because goal-directed care would elevate the importance of understanding each person as a unique human being, it would strengthen relationships and reduce prejudices that contribute to health care disparities. 

Similarly, if the outcomes of interest were defined from the perspective of patients, goal-directed care would almost certainly improve outcomes, since they would be the direct focus of care rather than an indirect consequence of problem-solving. And because patients would have greater involvement in determining health improvement strategies, they would be more likely to implement them.

A goal-directed approach could also be expected to increase patient satisfaction since the values, preferences, knowledge and opinions that each patient brings to the doctor-patient relationship would be more highly valued. Discussions would focus directly on patient goals. This would strengthen the relationship and support patient's sense of competence and autonomy.

Most important to current reform efforts, goal-directed care would be less expensive than problem-oriented care. Focusing on prevention of premature death and disability would increase delivery of high impact primary preventive services. More strategic and timely interventions would reduce tests and treatments. The growth and development goal would put a greater emphasis on natural healing processes. That would mean, for example, fewer antibiotics for self-limited infections.

Direct efforts to improve functional abilities could have indirect economic benefits as well, including increased productivity of patients at work, school, and home. And because of the “good death” goal, most people would have completed advance directives, preventing many unwanted interventions at the end of life. Finally, closer relationships between doctors and their patients could reduce the number of malpractice suits, lowering insurance premiums over time while reducing the practice of defensive medicine.

### Measurement

Value is quality divided by cost. In the case of health care, quality typically includes both quality of care and desirable outcomes. The measures of quality and cost should reflect the mission and goals of the system being evaluated. If the mission of the health care system is to provide help that is actually helpful, only when needed, to people striving to derive maximum pleasure and value from life’s journey, then it would make sense for the measures of value to relate to the four major goals discussed above.

While measuring prevention of premature death and disability is not as easy as measuring A1c levels, it also is not impossible. It is possible, with sufficient accuracy, to estimate life and health expectancies based upon individual risk factor profiles [30]. An indication of higher value care would be implementation of higher value preventive services, those with the biggest impact on life and health expectancy (process) and change in life expectancy relative to no preventive interventions.

Measurement of quality of life involves a different set of challenges. Since the outcome of interest would be different for each person, measurement would require individualizable assessment tools. The process of care measures could include documentation and discussion of meaningful life activities and implementation of appropriate strategies to preserve or enhance them. Goal achievement (outcome) could be assessed using individualizable quality of life tools, several of which have been developed.

Determination of the proportion of people who are likely to experience a good death could be as simple as determining the rates of the end of life discussions with health care professionals and completion of advance directives (process). In addition, it should be possible to query family members and surrogates at an appropriate interval following a person’s death to assess whether their wishes were followed (outcome).

Growth and development are measured in children. It is, therefore, reasonable to assume that appropriate metrics could be developed for adults. We would then need to clarify the appropriate roles for the health care system in promoting physical, psychological and spiritual growth (process) and ways to measure progress toward achieving their growth and development goals (outcome).

Patient satisfaction is routinely used as a health care outcome measure. Aside from the obvious problems of ceiling effect and socio-demographic biases, it is a reasonable measure of accessibility, cleanliness, convenience, staff courtesy, physician attentiveness, and concern, etc. However, it is not a good measure of the knowledge and insights clinicians contribute to the health care process.

A goal-directed approach should improve adherence to therapeutic plans since those plans would be constructed collaboratively and directed toward agreed upon goals. In fact, since adherence is related to both quality of care and outcomes, it might be a good measure for use in value calculations.

Measurement of the cost is beyond the scope of this discussion. The only comment that I would like to make is that in addition to traditional calculations of cost, it might be helpful to estimate the average cost per individual over the course of their life, in order to take into account the long-term effects of prevention and to account for expenditures across payer sources. 

What about the measurement of health? Defined health as “deriving value from life’s journey,” makes it a verb not a noun, a process not a state, the slope of a curve not a point. Based upon that definition, it would be possible to become healthier at any age. However, because health would involve at least four components, each with lots of subcomponents, it would be hard to measure in two dimensions. But as many wise people have said, the most important things can’t be measured.

### Obstacles and opportunities

Despite the simple logic and potential advantages of goal-directed health care, broad adoption and implementation would not be easy. Problem-oriented care is built into the recruitment and selection of health care professionals, their subsequent training, the record systems they are required to use and the way that they are evaluated and paid. The entire language and culture of health care is based on problem-oriented thinking and as we know, semiotics is often more important than systems. In addition, the current clinical knowledge base was built upon and organized around problems and the methods used to discover new information are designed similarly. If the health care system suddenly switched from problem-oriented to goal-directed care, clinicians and patients would find that much of the information they would want is not available or has not been analyzed in such a way as to be helpful.

Despite these formidable obstacles, there are some signs that such a transformation could happen, and in fact, is happening to some degree. Newer areas of clinical care such as Hospice Care, Sports Medicine, Geriatrics and Integrative and Complementary Medicine seem to be more goal-directed. The latest clinical practice guidelines for cholesterol management and prophylactic low-dose aspirin are based on the risk of cardiovascular events rather than cholesterol. The term "patient-centered" has entered the general lexicon. Patient advisory and patient advocacy groups are increasing in number and influence. Medicare now pays for health risk assessments and for end-of-life discussions. 

However, well-established paradigms are extraordinarily resilient. It is, therefore, unlikely that a real change in approach will occur until several somewhat predictable things happen concurrently. Those things are most likely to include the rapid evolution of genomics, the renewed escalation of health care costs despite current cost control measures and the growing dissatisfaction of clinicians and their patients with the current approach.

## Conclusions

Our current problem-based definition of health and our problem-oriented approach to health care are likely to continue to make it difficult to improve quality of care and to reduce health care costs. Redefining health as the effort to derive pleasure and value from life’s journey suggests that the purpose of health care is to help each person achieve four major goals: prevention of premature death and disability, maintenance and enhancement of quality of life, personal growth and development and a good death. A goal-directed approach to health care system would tie interventions directly to meaningful outcomes and provide a framework for prioritization. It would also enhance therapeutic relationships, improving adherence to therapeutic plans and reducing the practice and cost of defensive medicine. As a result, health care would be more effective, less expensive, and more humane.
